# Biomechanical-Based Protocol for *in vitro* Study of Cartilage Response to Cyclic Loading: A Proof-of-Concept in Knee Osteoarthritis

**DOI:** 10.3389/fbioe.2021.634327

**Published:** 2021-05-03

**Authors:** Paolo Caravaggi, Elisa Assirelli, Andrea Ensini, Maurizio Ortolani, Erminia Mariani, Alberto Leardini, Simona Neri, Claudio Belvedere

**Affiliations:** ^1^Movement Analysis Laboratory, Istituto di Ricovero e Cura a Carattere Scientifico (IRCCS) Istituto Ortopedico Rizzoli, Bologna, Italy; ^2^Laboratory of Immunorheumatology and Tissue Regeneration, Istituto di Ricovero e Cura a Carattere Scientifico (IRCCS) Istituto Ortopedico Rizzoli, Bologna, Italy; ^3^I Orthopaedic and Traumatologic Clinic, Istituto di Ricovero e Cura a Carattere Scientifico (IRCCS) Istituto Ortopedico Rizzoli, Bologna, Italy; ^4^Department of Medical and Surgical Sciences, Alma Mater Studiorum-Università di Bologna, Bologna, Italy

**Keywords:** osteoarthritis, knee cartilage, chondrocyte mechanotransduction, knee biomechanics, knee loading

## Abstract

Osteoarthritis (OA) is an evolving disease and a major cause of pain and impaired mobility. A deeper understanding of cartilage metabolism in response to loading is critical to achieve greater insight into OA mechanisms. While physiological joint loading helps maintain cartilage integrity, reduced or excessive loading have catabolic effects. The main scope of this study is to present an original methodology potentially capable to elucidate the effect of cyclic joint loading on cartilage metabolism, to identify mechanisms involved in preventing or slowing down OA progression, and to provide preliminary data on its application. In the proposed protocol, the combination of biomechanical data and medical imaging are integrated with molecular information about chondrocyte mechanotransduction and tissue homeostasis. The protocol appears to be flexible and suitable to analyze human OA knee cartilage explants, with different degrees of degeneration, undergoing *ex vivo* realistic cyclic joint loading estimated via gait analysis in patients simulating mild activities of daily living. The modulation of molecules involved in cartilage homeostasis, mechanotransduction, inflammation, pain and wound healing can be analyzed in chondrocytes and culture supernatants. A thorough analysis performed with the proposed methodology, combining *in vivo* functional biomechanical evaluations with *ex vivo* molecular assessments is expected to provide new insights on the beneficial effects of physiological loading and contribute to the design and optimization of non-pharmacological treatments limiting OA progression.

## Introduction

Osteoarthritis (OA) is considered the sixth-leading cause of disability in the world (Kloppenburg and Berenbaum, 2020). It is an evolving disease and a major cause of impaired mobility leading to an important reduction of the quality of life and increased costs on healthcare systems (World Health Organization, 2003; Piscitelli et al., 2012). Since OA is non-reversible, its prevalence increases significantly with age, although with differences between genders. In particular, worldwide estimates report that 9.6% of male and 18% of females aged above 60 years show symptomatic OA (Woolf and Pfleger, 2003; World Health Organization, 2003). Weight-bearing joints, such as the hip and knee, are the most affected anatomical structures. In particular, knee OA was ranked the 11th highest contributor to global disability showing a global age-standardized prevalence of 3.8% (Cross et al., 2014). While walking and exercise therapy are considered effective for pain relief (Huang et al., 2003; Beckwee et al., 2013; Juhl et al., 2014; Fransen et al., 2015) and are generally recommended (Fernandes et al., 2013; Bruyere et al., 2014; McAlindon et al., 2014; Nelson et al., 2014), most patients suffering from severe OA will eventually need surgical treatments. Though partial- or total-joint-replacement become necessary for pain relief and for restoring the original joint function, they involve complex invasive surgical procedures with high social costs. As far as the knee, its complex joint mechanics makes the outcome of surgical procedures not always fully satisfying, as a number of patient-specific anatomical and kinematic factors can affect duration of the implant (Ensini et al., 2012), which may require revision surgery over time. In the European Union, OA has been reported to be the main cause for surgical intervention (Merx et al., 2003).

In this context, a deeper understanding of the joint cartilage wear patterns is of paramount importance to achieve greater insight into the main causes of knee OA (Andriacchi et al., 2004). The knee has a rather complex movement with respect to the simple ball-and-socket hip joint. The lateral femoral condyle presents a pronounced posterior translation during knee flexion, which is consistent with the simultaneous internal tibial rotation; this has been referred to as a medial-pivoting motion (Asano et al., 2001). This combination of rolling and sliding motion is unique in the human body and, combined to the large loads acting on the knee in some of the daily living activities (Kutzner et al., 2010; Qi et al., 2013), can result in permanent damages to OA-prone knee cartilage. The analysis of the main biomechanical factors affecting knee cartilage loading and biological response is critical to identify effective conservative treatments capable to delay OA progression and related surgeries. Among the most important factors affecting knee OA are the severity of the degenerative changes due to biomechanical factors, such as the lower limb mechanical axis and abnormal knee joint kinematics (Moschella et al., 2006). In this perspective, a thorough *in vivo* biomechanical analysis to estimate the knee internal joint forces and contact areas during locomotion is essential to fully understand knee functioning (Hinterwimmer et al., 2005; Berti et al., 2006; Edwards et al., 2008; Morimoto et al., 2009; Hosseini et al., 2010; Scheys et al., 2013; Battaglia et al., 2014). This has been conducted mostly *in vivo* using gait analysis, though mainly for hip and knee joint replacements (Belvedere et al., 2014). The still-unclear association between molecular cartilage homeostasis and effects of cycling joint loading during motor activities requires further investigation to achieve better understanding of OA pathomechanics. Filling this gap becomes essential for efficient OA management. In order to counteract the irreversibility of OA, a reasonable medical approach may be focused on the prevention of this disease or, at least, on slowing down its progression.

By the biological point of view, accumulating literature evidence points to the ambivalent effect of joint loading on cartilage: physiologic loading promotes cartilage anabolism whereas reduced or excessive loading stimulate tissue catabolism (Leong et al., 2011; Sun et al., 2011). Duration, frequency and magnitude of the load applied to the articular cartilage are critical for tissue homeostasis and can stimulate matrix-protein remodeling and biosynthesis, improve mechanical properties and sustain chondrocyte differentiation (Sun, 2010). The underlying mechanisms are only partially elucidated, mostly on *in vitro* or animal models, whereas, to our knowledge, few studies exist on *ex vivo* dynamic stimulation of human cartilage tissue (Huang et al., 2007; Bougault et al., 2008; Haudenschild et al., 2010; Ramachandran et al., 2011; Jeon et al., 2012; Kawakita et al., 2012; Griebel et al., 2013; Bougault et al., 2014). A variety of cellular structures and cell surface molecules including integrins, cell adhesion receptors, and ion channels have been implicated in mechanotransduction in cartilage (O’Conor et al., 2014). Moreover, cartilage extracellular matrix components and matrix enzymes play a pivotal role in cartilage response to mechanotransduction. In a recent study we demonstrated that compression of OA human cartilage modulates the inflammatory milieu by differently affecting the expression of components and homeostasis regulators of cartilage extracellular matrix (Dolzani et al., 2019). Future challenges include better characterization of the signaling and transcriptional pathways involved and identification of the molecular mechanisms that differentiate degenerative responses from protective and beneficial effects of exercise treatments (demonstrated by clinical evidence) (Abramoff and Caldera, 2020).

The main scope of this study is to offer an original investigation methodology in mechanotransduction for the *in vitro* testing of human explanted knee cartilage tissue based on physiological biomechanical data, and according to cartilage scoring. A feasibility proof of the proposed methodological protocol has been here originally performed as concerning the extraction of molecular information about chondrocyte mechanotransduction and tissue homeostasis by high throughput gene expression analysis.

## Proposed Investigation Protocol

**FIGURE 1:**
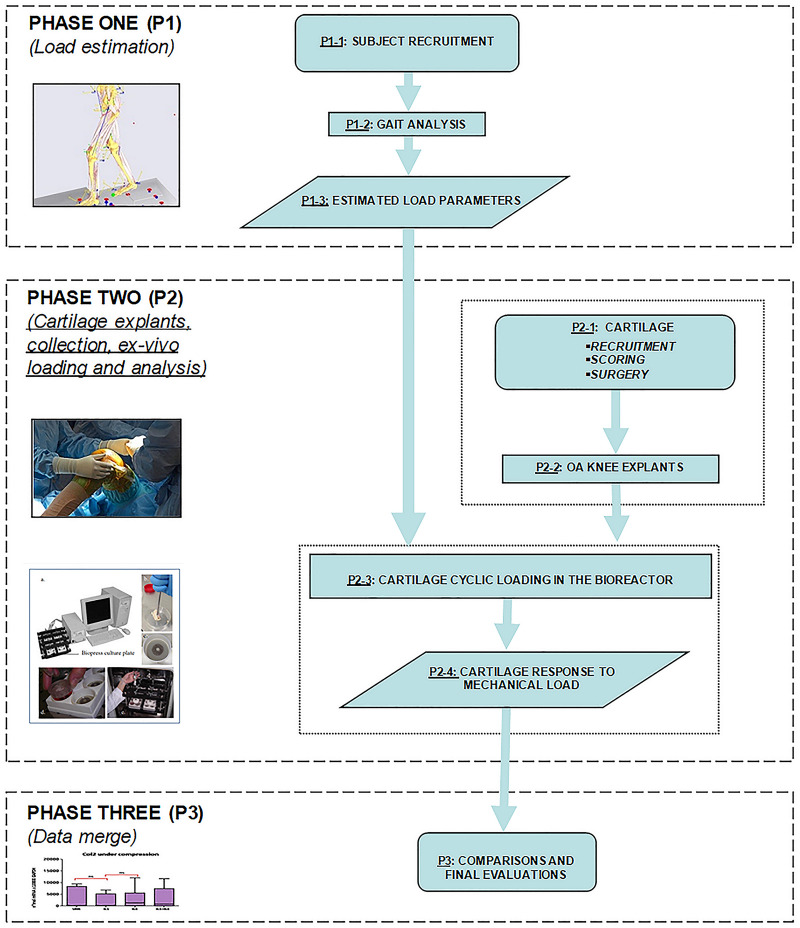
Proposed Investigation Workflow reporting the sequence of all phases.

The main activities of the proposed investigation protocol on mechanotransduction for *in vitro* testing of human explanted knee cartilage are summed up in Figure 1.

In the first phase (P1), a biomechanical analysis is performed on a defined number of subjects (P1-1), healthy or suffering from knee OA, depending on the project plan. Biomechanical data recruitment is essential to estimate the magnitude and direction of physiological joint loading, during the execution of motor tasks of daily living, using a combination of kinematic analysis via stereo-photogrammetry, and detailed kinetic analysis of the ground reaction force via force and pressure plates (P1-2). Lower limb kinematic data and measured external forces during mild motor activities or exercises can be applied to a model of the knee joint to estimate the internal forces and moments (P1-3). In this phase, the investigator can take advantage of several supporting data reported in the relevant literature and briefly summed up here below. Kinematic and kinetic data are intended to set up an effective and realistic loading regime to be applied *ex vivo* to cartilage explants. In particular, the estimated axial component of the internal loading on the distal femur are converted into the corresponding compression values to be applied *ex vivo*, in order to obtain a more precise simulation of the mechanical stimulation experienced by the knee articular cartilage *in vivo*. In accordance with the biomechanical analysis, different loading regimes can be applied to the cartilage samples.

In the second phase (P2) cartilage (P2-1) is recruited after appropriate macroscopic scoring, possibly including also inspections based on MRI. In detail, OA cartilage samples can be obtained from OA patients undergoing knee surgery, such as total knee replacement. Collection of healthy cartilage is a demanding task, due to possible ethical and regulatory issues specific for each single country. For example, cartilage samples can be obtained from oncological patients subjected to surgical removal at the knee level, under appropriate inclusion and exclusion criteria. Image-based inspection is helpful to characterize the areas of the knee with different cartilage alteration. The latter can be performed via routine MRI-based protocol with additional sagittal T1rho and T2 sequences and mapping. MRI images from all examinations must be reviewed by expert musculoskeletal radiologists to determine the presence, the extent, and the features of cartilage lesions on the femoral articular surfaces. To further enhance the precise identification of the area of cartilage explantation, and for geometric characterization of the whole articular cartilage under testing, the surgeon can take advantage of state-of-the-art systems for computer-aided knee-based surgery (Belvedere et al., 2014). Moreover, cartilage degeneration is visually inspected by the surgeon, attributing a macroscopic score that will be subsequently confirmed by microscopic scoring through histological evaluation. The biological assessment of the explants is always performed in P2. Cylinders of cartilage are cut with a coring tool from the femoral condyles, and then grouped according to both the original anatomical location, and to the degree of cartilage degeneration (based on MRI and/or macroscopic scores). The cartilage samples are then exposed to *ex vivo* cyclic loading in a bioreactor (P2-3), in agreement with the estimated *in vivo* knee joint loads. Subsequently, the corresponding cartilage response to the mechanical load is evaluated (P2-4) in terms of modulation of cartilage homeostasis marker expression. Cartilage samples and culture supernatants are recovered for downstream analyses, including morphological analyses on explants (histology, immunohistochemistry), molecular analyses on explants (RNA extraction followed by gene expression analysis) or on supernatants (protein expression analysis). Data obtained from compressed samples are compared to the same paired samples (same topographical areas and same score) maintained in unloaded conditions.

In the last phase (P3), all obtained data are meant to be merged for overall comparison and final evaluations.

## Materials and Methods

In the following sections we are reporting the assumptions made for the application of the different steps of the proposed protocol. A pilot application to test the feasibility of high throughput gene expression analysis is also presented.

### Estimation of Knee Joint Loading (Phase P1)

Knowledge of the forces and moments acting at the knee during common motor activities, along with information about the tibio-femoral contact areas, are essential to identify reference input data (e.g., pressure, axial load, load frequency) to perform corresponding knee cartilage stress analysis using *ad-hoc* devices such as a bioreactor.

Measuring physiological knee joint loading *in vivo* is complex, and most of the data reported in the literature are based on musculoskeletal (McLean et al., 2003; Fregly et al., 2012) or EMG-driven modeling approaches (Buchanan et al., 2004). Two studies by some of the authors of the present study (D’Angeli et al., 2014) were aimed at contributing to this knowledge using an original non-invasive methodology. Gait analysis and 3D anatomical-based data were recorded in 20 healthy young volunteers and used to estimate the moments about the femoral and the tibial shaft during common motor activities (e.g., level walking, squatting, stair ascending/descending). While an instrumented knee prosthesis has also been proposed for direct measurement of internal knee forces (D’Lima et al., 2006), the largest collection of knee joint loading data is from the open OrthoLoad database (Julius Wolff Institute and Charité – Universitätsmedizin Berlin, 2020). An instrumented knee prosthesis with telemetric data transmission allowed to measure axial (compression), medio-lateral and antero-posterior (shear) forces between femoral and tibial components in several patients and for several activities of daily living (Heinlein et al., 2009). The combination of knee loading data and knee joint contact areas allows to estimate the pressure exerted at the tibio-femoral joint. As far as the latter, Table 1 is reporting a critical review of the relevant literature by taking into account only those studies reporting information on the methodological approach, sample size, tibio-femoral joint flexion angle and loading conditions, and differentiation between medial and lateral knee compartments. Studies differ in terms of axial loading applied to the knee, knee flexion angle, instruments for force estimation, and *in vivo* or *in vitro* approach. The reported average knee medial compartment contact area ranges from 235 to 670 mm^2^ across all studies. Liu et al. (2010) reported the most complete dataset measured *in vivo* using 3D video-fluoroscopy and MRI in 8 subjects (age 32–49 years; average BMI 23.5 kg/m^2^), including medial compartment contact area at five gait time-points.

**TABLE 1:** Knee joint contact areas as from a critical literature review.

Studies	Technique	Sample size	External load (N) applied	Knee flexion angle (°)	Contact area (mm^2^)	
					Medial compartment	Lateral compartment
Fukubayashi and Kurosawa (1980)	*In vitro*, sensor sheets	7	500	0	240	160
Henderson et al. (2011)	*In vivo*, MRI	1	365	Full extension in weight bearing	511 ± 143.1	256 ± 40.3
Hinterwimmer et al. (2005)	*In vivo*, MRI	12	0	0	487 ± 103	220 ± 72
				30	404 ± 98	299 ± 58
				90	302 ± 79	255 ± 51
Hosseini et al. (2010)	*In vivo*, MRI combined with video-fluoroscopy analysis during single-leg upright standing for 300 s	6	Full body weight	Full extension in weight bearing	From 47.0 ± 21.2 (at 0 s) to 263.2 ± 19.6 (at 300 s)	From 20.3 ± 20.0 (at 0 s) to 135.6 ± 20.8 (at 300 s)
Kettelkamp and Jacobs	*In vitro*, roentgenography	14	23–79	0–35	480	300
Liu et al. (2010)	*In vivo*, MRI combined with gait and 3D video-fluoroscopy analysis during a standard gait cycle	8	Not reported	Heel strike	235 ± 11	200 ± 84
				30%	467 ± 61	411 ± 159
				50%	354 ± 97	329 ± 97
				80%	428 ± 87	451 ± 109
				Toe-off	260 ± 140	331 ± 167
Morimoto et al. (2009)	*In vitro*, axial testing machine and pressure-sensitive film	22	1,000	0	578.3 ± 177.0 (3.6 ± 0.4)^#^	443.1 ± 121.0 (3.9 ± 0.5)^#^
				15	488.5 ± 140.4 (3.5 ± 0.3)^#^	495.3 ± 146.4 (3.6 ± 0.4)^#^
				30	449.9 ± 151.4 (3.4 ± 0.3)^#^	507.0 ± 189.2 (3.7 ± 0.4)^#^
				65	468.4 ± 143.1 (3.7 ± 0.5)^#^	507.8 ± 152.6 (3.6 ± 0.6)^#^
Patel et al. (2004)	*In vivo*, MRI	10	133	60	374	276
Périé and Hobatho (1998)	*In vivo*, MRI and FEM	1	0	Full extension	293 ± 72	111 ± 57
Yao et al. (2008)	*In vitro*, MRI	10	0	−4	670 ± 140*	600 ± 80*
				134	560 ± 110*	375 ± 75*

*Average data including contact with the meniscus. ^#^In brackets corresponding measured mean pressure data in MP.

While standard gait-analysis instrumentation does not allow to measure *in vivo* joint loading, this has been found to be significantly correlated to the knee external adduction moment (Kutzner et al., 2013), which is commonly measured via force plate measurements and knee kinematics. A linear relationship between the medial tibio-femoral contact force (Fmed) and the External Knee Adduction Moment (EAM) during stance phase duration has been inferred using data from 9 subjects (age 70 ± 5 years; BMI = 30.6 ± 4.6 kg/m^2^) (Kutzner et al., 2013):

F⁢m⁢e⁢d=100+26*E⁢A⁢M

where Fmed is reported in % of Body Weight (%BW), and EAM in % of BW^∗^height.

**FIGURE 2:**
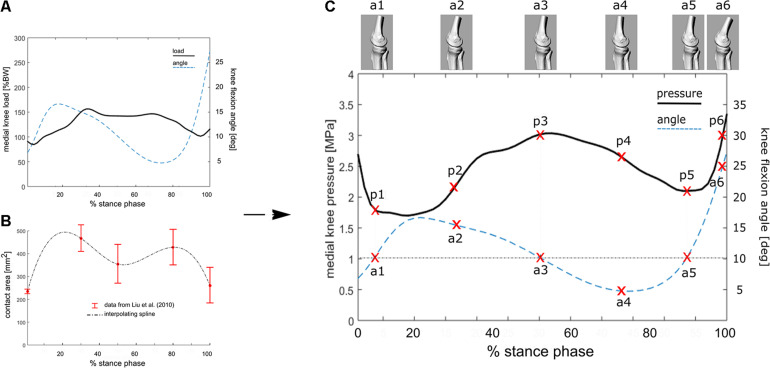
**(A)** Knee flexion angle and the estimated internal tibio-femoral joint forces during stance phase of walking using the linear relationship between EAM and knee forces reported in the equation. EAM and knee kinematics were calculated as the average across five walking trials for a subject with knee OA. **(B)** Interpolated temporal profile of cartilage contact area over stance phase duration, using data from Liu et al. (2010). **(C)** Estimated temporal profile of tibio-femoral contact pressure over stance phase of walking for the knee OA patient; the curve is obtained by dividing the contact force **(A)** by the contact area **(B)**. 3D representations of the tibio-femoral joint angle (a1–a6) at different time frames are shown. The knee is flexed 10° at three times of the of the stance phase, while the relevant cartilage region is subjected to pressure p1, p3 and p5.

This relationship (*R*^2^ = 0.56 and RMS error = 0.28^∗^BW, in Kutzner et al., 2013) has been used here to estimate the average knee loading (Figure 2A) in an exemplary patient (M; age 52 years; weight 73 kg; height 1.73 m; Body Mass Index 24.4) suffering from knee OA. Average tibio-femoral joint flexion angles and adduction/abduction moments were obtained from 5 walking trials recorded during comfortable walking-speed at 100 Hz (Figures 2A,C). Data were obtained using a validated lower limb skin-marker based kinematic protocol (Leardini et al., 2007) and angles calculated according to the joint coordinate system (Grood and Suntay, 1983; Cappozzo et al., 1995), as recommended by the International Society of Biomechanics. By assuming the knee contact area to vary continuously and smoothly over stance duration—which is acceptable for most time-dependent biomechanical and biological variables—the mean temporal profile of the knee contact area was approximated by a cubic spline interpolating the five contact-area points (Figure 2B) reported in Liu et al. (2010). Pressure at the knee medial compartment (Figure 2C) was estimated by dividing the knee contact force (Figure 2A) by the contact area (Figure 2B) across normalized stance-phase duration.

### Cartilage Explants Collection, *ex vivo* Loading and Analysis (Phase P2 and P3)

#### Sample Recruitment

**FIGURE 3:**
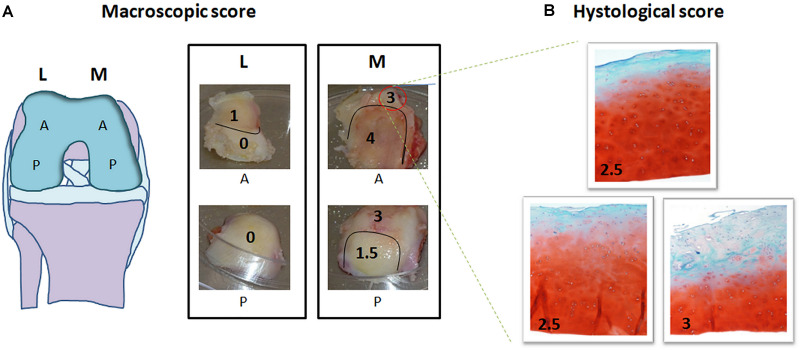
Macroscopic and histological scoring of femoral condyles from a representative mono-compartmental knee OA patient. **(A)** Schematic drawing of the knee and images of the four condyle areas: femoral condyles (medial, M and lateral, L) removed by the surgeon were divided each in anterior (A) and posterior (P). Each of the four areas was scored by the surgeon based on macroscopic degeneration of the cartilage tissue by Collins grading system, thus obtaining further zoning. **(B)** For each zone identified, histological evaluation was performed in triplicate in three subareas. Macroscopic and histological scores attributed are indicated in each picture, histological images being at 10× magnification.

In order to test the protocol, 9 mono-compartmental knee OA cases (2 F, 7 M; age ± SD: 69.6 ± 8.7 years; mean BMI ± SD: 28.0 ± 4.2 kg/m^2^) were recruited. Out of these, 6 cases (2 F, 4 M; age ± SD: 69.6 ± 9.8 were used to check for macroscopic scoring reliability and 3 cases (3 M; age ± SD: 74.3 ± 6.0 years; BMI: 24.2; 26.6; and 32 kg/m^2^, respectively) were used to test the feasibility of high throughput gene expression analysis. The study was carried out in compliance with the Helsinki declaration, and approved by the local Ethic Committee (CE-AVEC Prot. Kneeload N. EM603-2018_89/2015/Sper/IOR_EM1), including documentation of written patient consent. Medial and lateral femoral condyles were collected at time of knee replacement surgery. For each knee condylar explant (medial and lateral) four topographical areas (corresponding to the original anatomical location and, therefore, exposed to different *in vivo* loading conditions) were identified: medial anterior, medial posterior, lateral anterior and lateral posterior (Figure 3A). Cartilage samples from six out of the nine recruited donors were processed for histology, while the samples from the remaining three donors were cored after subchondral bone removal. Obtained fresh cartilage cylinders were cultured overnight and then exposed to *ex vivo* controlled compression (Supplementary Tables 1, 2).

#### Macro- and Microscopic Cartilage Scoring

The surgeon in charge for total knee replacement attributed macroscopic scoring to the cartilage areas according to the Collins grading system of disease severity (from 0 to 4, with 0 corresponding to apparently normal cartilage, and 4 corresponding to completely degenerated tissue) (Pritzker et al., 2006). Each of the four condylar topographical areas (lateral anterior, lateral posterior, medial anterior and medial posterior) can be homogeneous, or further zoned in sub-areas, according to the macroscopic score (Figure 3A). Samples of full thickness cartilage were freshly recovered from all identified sub-areas and scored after conventional Safranin O-staining. Briefly, 5 μm thick sections of FFPE samples were rehydrated and stained with Haematoxylin/Eosin, 0.1% Safranin-O and 0.02% Fast Green (SIGMA-ALDRICH, Munich, Germany). Three representative slices from each sub-area (homogeneous for macroscopic score and anatomical position) were independently analyzed by two experienced biologists for histopathology grading score attribution following the OARSI criteria (from grade 0, corresponding to intact cartilage, to grade 6, corresponding to complete cartilage degradation and bone deformation). The mean of three different positions for each sub-area was recorded (Pritzker et al., 2006; Figure 3B). Evaluations were performed with an Eclipse 90i microscope and NIS elements software (NIKON CORPORATION, Tokyo, Japan).

Pearson’s correlation was used to compare macroscopic and microscopic scores from 22 samples (areas with homogeneous macroscopic score obtained from 6 donors). The macroscopic score provided by the surgeon was compared to the microscopic mean score of three representative slices for each of the 22 areas (Supplementary Table 1).

#### Controlled Cartilage Compression

Within phase P2 the knee loading parameters estimated in Phase P1 are applied to fresh cartilage samples. To evaluate the effect of mechanical compression on articular OA cartilage in relationship to the level of cartilage degeneration, samples from different topographical areas and with different macroscopic score are separately recovered, then cylinders of 2.5 mm diameter cartilage tissue are cored with a biopsy needle. Cartilage explants in D-MEM medium (SIGMA, Sigma Aldrich, St. Louis, United States) are serum starved for 24 h, then exposed to unconfined controlled compression in a computer-regulated bioreactor (FlexCell FX-4000C, Flexcell International Corporation, United States). The system allows for control of duration, frequency and intensity of the applied load in order to simulate different motor tasks and to span physiologic and extra-physiologic stimulations. Compression regimes are performed in an incubator under controlled temperature (37°C) and CO_2_ level (5%). The assembly of the compression unit and sample accommodation in the compression well has been described elsewhere (Dolzani et al., 2019). Multiple loading positions in compressed plates allow for simultaneous compression of paired samples in basal or stimulated conditions (such as in the presence of pro- or anti-inflammatory mediators like IL-1β and IL-4, respectively) (Dolzani et al., 2019). After compression, fresh tissue samples can be dedicated to histological, immunohistochemical and/or molecular analyses that can be performed individually or in parallel, the only limit being the available tissue.

As exemplary application of the above-described protocol, here we run a compression experiment on three knee OA cases using the estimated knee loading parameters during normal walking (1 Hz loading sinusoid). Fresh cartilage samples were cored and cartilage cylinders with homogeneous macroscopic degeneration score (0–1) were serum starved overnight in the incubator, then exposed to loading. Compression was implemented by setting the flexcell apparatus with a force sinusoid—1 Hz frequency—for 45 min, applied to the superior surface of a single cartilage cylinder per well, vertically arranged as to reproduce the cyclic pressure established in the biomechanical evaluation (3 MPa). Paired uncompressed samples were used as controls.

#### Array-Based Gene Expression Analysis

Molecular analyses were performed on total RNA by array-based gene expression analysis to provide proof of concept of applicability of this kind of high throughput analysis to these challenging samples. We performed a pilot experiment on cartilage cylinders from three donors. Cartilage explants from areas with macroscopic score corresponding to 0–1 were used. Cartilage explants are cylinders of 2.5 mm diameter and variable height (ranging from 1 to 3–4 mm) and weight (ranging from about 20 to about 40 mg) depending on tissue zonal thickness and degeneration. Two uncompressed/unstimulated samples were run in duplicate to test for assay reproducibility, while two samples were also analyzed after compression and in absence or presence of IL1-β stimulation (2 ng/ml). Cartilage areas and donors used for these analyses are specified in Supplementary Table 2.

Fresh explants were immediately recovered and liquid nitrogen frozen, then pulverized using the Mikro-Dismembrator S grinding mill (Sartorius Stedim Italy SpA, Italy) in 5 ml PFTE shaking flasks with a stainless-steel grinding ball (2,000 rpm, 45″). Total cellular RNA was purified from pulverized explants with Trizol isolation reagent (Thermo Scientific, Germany) and spectrophotometric quantification was performed (mean recovery 2.5 μg, range 0.8–5.4 μg). Total RNA was treated with the RNase-Free DNase Set (Qiagen, Germany), cleaned-up in RNeasy mini columns (Qiagen) following manufacturer’s instructions to deplete contaminating DNA and reverse transcribed by the RT2 First strand kit (Qiagen). The expression of a focused panel of 84 genes involved in wound healing together with five housekeeping genes (actin beta-ACTB; Beta-2-microglobulin-B2M; Glyceraldehyde-3-phosphate dehydrogenase-GAPDH; Hypoxanthine phosphoribosyltransferase 1-HPRT1; Ribosomal protein large P0-RPLP0) was evaluated by the RT2 Profiler PCR Array PAHS-121Z (Qiagen) in a Rotor-gene 6000 real-time analyzer (Corbett, Concorde, NSW, Australia), following manufacturer’s instructions. The system includes genomic DNA control, reverse transcription control and positive and negative controls.

The threshold cycle (Ct) values for all the genes were calculated by the software of the real time PCR instrumentation and considered as a negative call when ≥35 or not detected. To compare Ct values across different runs, a single baseline within the exponential growth phase of the reaction curves was manually set. Relative gene expressions were calculated by the ΔΔCt method relative to the housekeeping genes showing Ct value consistency (ACTB; B2M; GAPDH; RPLP0). Differences between groups were analyzed by the Web-based PCR Array Data Analysis Software (SABiosciences^^TM^, Frederick, MD) available at https://dataanalysis.qiagen.com/pcr/arrayanalysis.php. Variations in gene expression in uncompressed vs. compressed samples were calculated by the ΔΔCt method, in which ΔCt is the difference between the gene of interest (GOI) Ct and the average of housekeeping gene (HKG) Ct. Fold-Change (2^–ΔΔCt^) is the ratio between the normalized gene expression (2^–ΔCt^) in the compressed sample and the normalized gene expression (2^–ΔCt^) in the uncompressed sample. A twofold change threshold for up- and down-regulation was considered. Genes were excluded from the fold change analysis if more than two conditions gave negative results.

## Results

### Estimated Knee Loading Conditions

**FIGURE 4:**
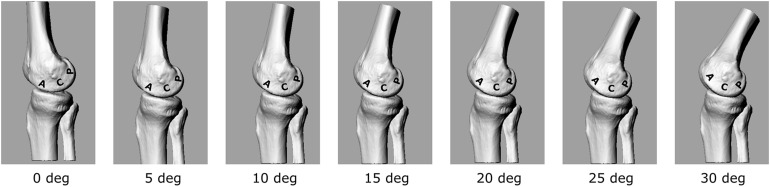
3D representation of the tibio-femoral joint at different knee flexion angles (0°–30°). A different cartilage region (Posterior, Central, Anterior) is loaded at each flexion angle.

The calculated EAM and knee flexion-extension angle for the knee OA patient analyzed here are generally consistent with physiological kinematic and kinetic data during walking. The knee shows a peak of flexion at heel strike, followed by continuous extension up to about 80% of stance, and flexion again prior to toe-off (Figure 2A). The shape of the interpolated temporal profile of cartilage contact area (Figure 2B) is consistent with that of the vertical ground reaction forces in walking, with two peaks at around 20 and 80% of the stance phase duration. These are also rather consistent with the estimated knee contact force shown in Figure 2A. Figure 2C is reporting the estimated pressure (MPa) at the knee medial compartment for the knee OA patient recruited in this study during gait. The pressure waveform varies between 1.5 and about 3.5 MPa over stance duration, showing two minima at around 20 and 80% of stance which is consistent with the larger contact area at these two time frames (Figure 2B). However, due to the knee flexion/extension motion in gait, a different joint cartilage region is subjected to a specific pressure at each time frame (Figure 4). Anterior and central cartilage regions may undergo multiple contacts during each walking cycle. For example, the anterior-central region of the cartilage compressed when the knee is flexed 10° (Figure 4), is subjected to pressure p1, p3 and p5, respectively, during the stance phase (Figure 2C). Whereas the cartilage in the posterior region, which is compressed for knee flexion angles larger than 20° (Figures 2C, 4), is loaded just once over stance duration.

The chosen input parameters used here for the bioreactor were those related to the posterior region cartilage region. Due also to the apparatus specifications, a sinusoid with 1 Hz frequency and amplitude of about 3 MPa was chosen to test the cartilage explants.

### Correlation Between Macro and Microscopic Scoring

**FIGURE 5:**
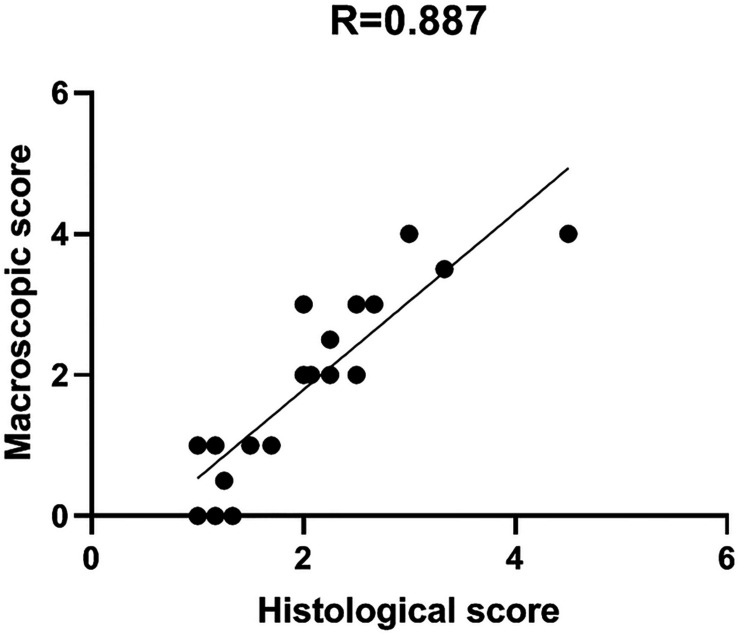
Macro and microscopic cartilage score correlation. Scatter plot representation of the correlation between macroscopic and histological scores for the evaluation of cartilage degeneration. Histological score is the mean of triplicate evaluations from the same area, performed by two independent researchers.

Comparison between macro and microscopic cartilage scoring was performed in samples from 6 donors to check for correlation between macroscopic scoring and actual cartilage degeneration, as assessed by histological evaluation. This provides evidence of correct assignment of samples to different groups. Macro and microscopic scores were compared by Pearson’s correlation analysis. Particularly, the correlation between Collins macroscopic score and OARSI histological score was evaluated on 22 different condyle areas from 6 OA donors (see Supplementary Table 1). Areas with completely degraded cartilage and subchondral bone exposure were excluded from the analysis due to the absence of cartilage tissue to be used for histology. The two scoring systems showed a high correlation coefficient *R* = 0.887, supporting the reliability and usefulness of the macroscopic score as criterion for cartilage area identification according to degeneration severity (Figure 5).

### Array-Based Gene Expression Analysis

To assess the feasibility of array-based gene expression approaches on limited amounts of OA cartilage exposed to mechanical compression, two cartilage cylinders per sample (from a single area per sample, macroscopic score = 0–1) were used for RNA extraction and gene expression pattern analysis of 84 human wound healing-related genes by RT-PCR arrays.

We firstly performed the analysis on unstimulated uncompressed samples from 2 OA patients to test the reproducibility of our protocol (Supplementary Table 2). Each sample was run in duplicate and the Ct variation coefficient (CV) of each analyzed gene was calculated. The average percentage CV of the two analyzed samples was 0.66 and 0.27%, respectively, indicating a very high reproducibility of our results. All internal quality controls met the requirements. Among the housekeeping genes included in the analysis, beta actin showed the higher stability.

To assess the effect of compression on wound healing-related gene expression we performed the same array-based analysis on 2 patients in basal conditions (uncompressed) and after compression. This was also done in presence of a pro-inflammatory stimulus (IL-1β). Sample used for this analysis are shown in Supplementary Table 2.

The applied compression regime corresponds to the estimated knee loading parameters during normal walking. Cartilage cylinders exposed to this unconfined compression were significantly deformed at the end of the loading regime (with a final reduction in height of about 60%).

**FIGURE 6:**
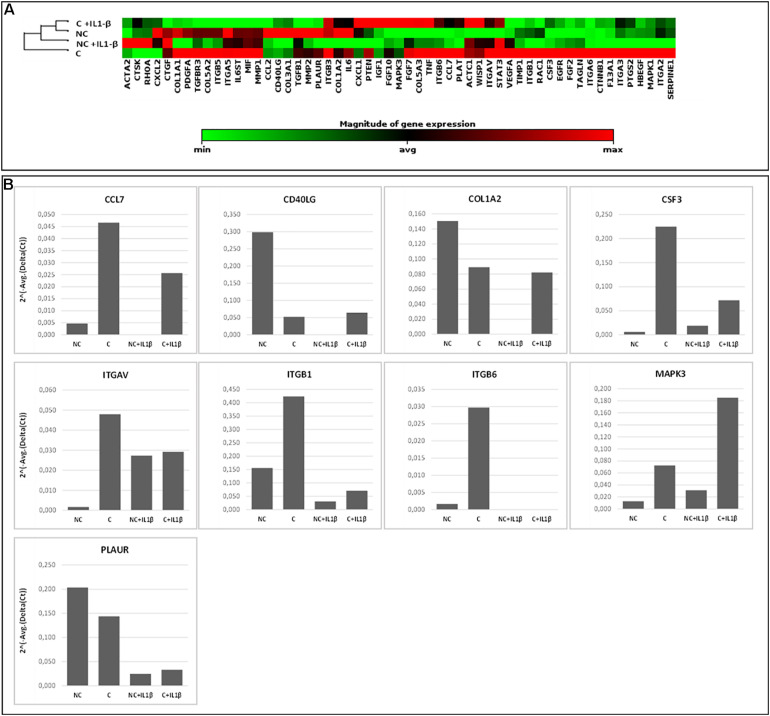
Array-based gene expression analysis of OA cartilage samples. A focused panel of 84 genes involved in wound healing was analyzed. Four experimental conditions were tested: (1) NC (not compressed cartilage, control group); (2) C (compressed cartilage); (3) NC+IL1-β (not compressed cartilage stimulated with the pro-inflammatory factor IL1-β); and (4) C+IL1-β (compressed cartilage stimulated with the pro-inflammatory factor IL1-β). Each group consists of two samples from two different donors. **(A)** Heatmap of the unsupervised hierarchical cluster analysis. Red indicates high levels of expression and green indicates low levels of expression; **(B)** Variations of selected genes after OA cartilage compression for 45 min at 1 Hz and 3 MPa and with addition of pro-inflammatory stimulation (IL1-β 2 ng/ml). Genes with a fold change of at least 5 in one out of 4 conditions are showed.

In Figure 6, gene expression analysis results are shown. The heatmap (Figure 6A) represents relative expression of the 55 expressed genes in the four experimental conditions (NC, C, NC+ IL1-β, C+IL1-β) as referred to the control group (NC) (see Supplementary Table 3 for detailed fold change values, while raw data are shown in Supplementary Table 4). Gene expression levels clearly differ after compression, both in unstimulated and in IL1-β-stimulated condition. Non-supervised hierarchical clustering of the four groups indicates that NC samples are more similar to C+IL1-β samples than the other groups. If considering a threshold fold change of at least 2 for both up and down-regulation, we observed that 89% of the genes were modulated by compression, 61% of them showing upregulation. Among the upregulated genes, Chemokine (C-C motif) ligand 7 (CCL7), CD40 ligand (CD40L), collagen 1 a2 (COL1A2), colony stimulating factor 3 (granulocyte) (CSF3), integrin alpha V (vitronectin receptor, alpha polypeptide, antigen CD51) (ITGAV), Integrin beta 1 (ITGB1), Integrin beta 6 (ITGB6), Mitogen-activated protein kinase 3 (MAPK3) and plasminogen receptor (PLAUR) appeared more than fivefold increased in compressed samples compared to controls. Array-based gene expression analysis is therefore feasible in the presented experimental model, despite the challenging tissue samples to be analyzed.

## Discussion

Mechanical stimulation plays a central role in the maintenance of cartilage homeostasis, and it is also involved in the pathogenesis of OA. The complex biological and mechanical scenario characterizing the knee joint homeostasis is generally based on several mechano-transduction mechanisms that elicit neutral, positive or negative biological responses in the subchondral bone and cartilage. Describing these mechanisms is a complex task for the number of biological and kinetic variables affecting this phenomenon. *In vitro* models of knee joint cartilage mechanotransduction are therefore particularly valuable as these allow to control the kinetic input, i.e., loading, and to measure the biological response under the same protocol conditions, and across a large population of homogeneous cartilage samples.

The present study aimed at proposing a novel methodological approach for the analysis of the knee cartilage response subjected to mechanical stimuli simulating common motor tasks of daily living. In particular, the theoretical loading parameters to be used as input for the bioreactor testing device were estimated by integrating the most comprehensive literature data on *in vivo* internal knee loading and cartilage contact area during gait. Gait data acquired in an exemplary patient suffering from knee OA were used to estimate theoretical loading parameters for the bioreactor. The superimposition of the temporal profiles of the *in vivo* knee sagittal-plane motion and cartilage pressure has allowed to reveal some interesting mechanical aspects which characterize the knee joint cartilage during walking. According to the anatomical region of interest, the cartilage appears to be subjected to different loading patterns, either in terms of loading frequency and/or magnitude. The anterior-central region of the cartilage, i.e., the region in contact from 0 to about 20° of knee flexion, is subjected to three pressure values (p1, p3, and p5) over the stance phase of walking. Conversely, the central-posterior region of the femoral cartilage (flexion angles larger than 20°) is subjected to just one pressure value over the stance phase. Therefore, in order to accurately simulate the *in vivo* tibio-femoral cartilage mechanics, it may be plausible to establish different cyclic pressure waveforms, both in terms of amplitude and frequency, according to the anatomical region the cartilage samples were explanted from. The amplitude of the theoretical cyclic pressure waveform would need to be set according to the location of the explants. In line with the kinetics of the exemplary OA patient used here, cartilage pressure ranges approximately between 1.5 and 3.5 MPa according to the stance time frame and thus to the cartilage location. In terms of cycle frequency, since the gait cycle time of comfortable walking is about 1 s, the bioreactor loading frequency could range between 1 Hz (for the posterior region cartilage samples) to about 5–10 Hz (for the central region cartilage samples).

Since the response of cartilage to loading is strictly dependent on the characteristics of the applied forces (frequency, intensity and duration) (Leong et al., 2011; Sun et al., 2011), these parameters need to be chosen and set with particular attention in order to reproduce the real forces occurring *in vivo*. In the proposed protocol, we applied compression loading to human cartilage explants *ex vivo* simulating the loading acting on the knee cartilage during walking following a thorough biomechanical analysis based on *in vivo* knee forces. Input parameters were run on a pilot case series of human OA cartilage cylinders in a bioreactor, differentiating samples according to tissue scoring and original anatomical position. In general, studies on human cartilage response to loading suffer from the reduced amount of available tissue and from the reduced number of cells normally present in the cartilage. Moreover, the anatomical and qualitative differentiation used here further reduces tissue availability for molecular analyses, thus representing the principal challenge to perform gene expression analysis in the presented experimental model. For this reason, high throughput multiple gene approaches (such as gene expression arrays) are likely to be a valuable tool to evaluate a significant number of molecules with reduced starting material. The drawback of such approaches is the need of higher quality RNA. Here we demonstrated that small cylinders of cartilage can be efficiently used for array-based gene expression analysis, giving reproducible results, and allowing to detect differences before and after compression. In the proposed protocol we were able to obtain sufficient amount of high-quality RNA to carry out the analysis of complex molecular pathways that can increase the understanding of the biomolecular mediators underlying the response to mechanical stimulation and possibly involved in cartilage integrity maintenance. In this proof-of-concept study, cartilage samples from two donors only were analyzed for gene expression, therefore attention should be paid before generalizing the results. We choose to analyze genes involved in the wound healing process to assess the effect of compression in cartilage tissue repair. Some of the genes most involved in mechanotransduction and regulated by compression forces are Integrins and the MAPK3 (Zhao et al., 2020), chemokines and factors involved in the inflammatory processes (CD40L, CCL7 and CSF3), the receptor for the matrix degradative enzyme plasminogen (PLAUR) and a component of cartilage matrix (COL1A2) (see Supplementary Table 3 for details on genes). As shown by non-supervised hierarchical clustering (Figure 6) NC samples are more similar to C+IL1-β samples than the other groups. Similarly to what observed in previous experiments (Dolzani et al., 2019), these results suggest a counteracting effect of compression against the pro-inflammatory stimulus.

The protocol proposed in this study can be applied to explore other molecular pathways implicated in the development of OA such as low-grade inflammation and innate immunity (Sokolove and Lepus, 2013; Silawal et al., 2018). Furthermore, in this study we hypothesized that chondrocytes from areas with distinct degrees of tissue alteration respond to loading differently. A very good correlation was obtained between macroscopic and microscopic scores. This confirms that a simple method such as macroscopic scoring, obtained by cartilage observation at the time of sample collection, could be a reliable method to quantify different degrees of degeneration.

The analysis conducted here to establish a set of biomechanical parameters to be used as loading inputs for a bioreactor should be interpreted with respect to some limitations. Although the relationship between EAM and internal knee forces used to estimate the knee force in our OA patient is rather accurate at early and mid-stance, it fails to accurately predict knee forces at late stance even considering the effect of covariates, such as walking speed and limb alignment (Kutzner et al., 2013). Thus, knee force and pressure temporal profiles should be considered to be reliable up to about 80% of stance phase, as the knee joint is almost fully unloaded at push-off. In addition, the temporal pattern of medial knee cartilage contact area over stance duration was estimated by interpolation of 5 points only. A more complete dataset of cartilage contact area throughout stance duration of walking should be sought to improve the accuracy in pressure estimation. In this proof-of concept study we validated different steps of the protocol with different samples without performing a complete workflow with a unique case series. We acknowledge that the characteristics and clinical history of the single knee OA patient recruited here, and thus the measured kinematic and kinetic data in gait, may not be fully representative of the average knee OA population. Specifically, the age of the OA patient (52 years) is lower than that of the *ex vivo* analysis cohort (69.6 ± 8.7 years); however, since the BMI (24.4 kg/m^2^) is within 1 SD of the mean in the cohort, we do not believe these differences compromise the generalizability of our results and significantly affect the conclusions. Ideally, a cohort of knee OA patients should be assessed via gait analysis to estimate the average physiological cartilage pressure and application frequency—according to the cartilage region; this data should be used as loading conditions in the bioreactor to test the explanted cartilage samples from the same cohort.

## Conclusion

In conclusion, we have here proposed an integrated protocol where *in vivo* estimated knee joint loading was used as compression regime applied *in vitro* to human cartilage. The molecular response to these stimulations has been analyzed to gain more insight into cartilage mechanobiology. A pilot application of this protocol to knee OA has also been presented. The combination of biomechanical and biological data may provide a significant added value to improve our understanding of knee cartilage mechanotransduction, by allowing to control the main parameters affecting the physiological condition. The proposed approach can be applied to different motor tasks (different compression regimes) or to specific cartilage samples (different anatomical regions or with different degrees of degeneration), and therefore could become a flexible tool to study different aspects of cartilage mechanotransduction.

## Data Availability Statement

The raw data supporting the conclusions of this article will be made available by the authors, without undue reservation.

## Ethics Statement

The studies involving human participants were reviewed and approved by CE-AVEC. The patients/participants provided their written informed consent to participate in this study.

## Author Contributions

EA, CB, PC, and SN: conceptualization, writing—original draft preparation, writing, review and editing, and project administration. EA, CB, PC, AE, and SN: investigation. AL and EM: resources and funding acquisition. EA, CB, PC, SN, and MO: data curation. EA, CB, PC, AE, SN, and MO: methodology. All authors have read and agreed to the published version of the manuscript.

## Conflict of Interest

The authors declare that the research was conducted in the absence of any commercial or financial relationships that could be construed as a potential conflict of interest.
