# Supplementary material for: Biomechanical-Based Protocol for in vitro Study of Cartilage Response to Cyclic Loading: A Proof-of-Concept in Knee Osteoarthritis
Source: Front Bioeng Biotechnol. 2021 May 3;9:634327. doi: 10.3389/fbioe.2021.634327 (PMC8126668; doi:10.3389/fbioe.2021.634327)
Supplement: Supplementary file 1 [file Data_Sheet_1.pdf]

SUPPLEMENTARY MATERIAL

Biomechanical-based protocol for *in vitro* study of cartilage response to cyclic loading: a proof-of-concept in knee osteoarthritis

Paolo Caravaggi[1], Elisa Assirelli[2], Andrea Ensini[3], Maurizio Ortolani[1], Erminia Mariani[2,4], Alberto Leardini[1], Simona Neri[2] and Claudio Belvedere[1]

[1] Movement Analysis Laboratory, IRCCS Istituto Ortopedico Rizzoli, Bologna, Italy: paolo.caravaggi@ior.it (P.C.); maurizio.ortolani@ior.it (M.O.); leardini@ior.it (A.L.); belvedere@ior.it (C.B.).

[2] Laboratory of Immunorheumatology and Tissue Regeneration, IRCCS Istituto Ortopedico Rizzoli, Bologna, Italy: elisa.assirelli@ior.it (E.A.); erminia.mariani@ior.it (E.M.); simona.neri@ior.it (S.N.).

[3] I Orthopaedic and Traumatologic Clinic, IRCCS Istituto Ortopedico Rizzoli, Bologna, Italy: ensiniandrea@gmail.com (A.E.).

[4] Department of Medical and Surgical Sciences, Alma Mater Studiorum-Università di Bologna, Bologna, Italy:

S1

**DONOR N. (1-9)**	**HISTOLOGICAL SCORE (0-6)**	**MACROSCOPIC SCORE (0-4)**
1	3.0	4.0
1	1.3	0.0
1	1.2	0.0
1	2.1	2.0
2	2.0	3.0
2	1.3	0.5
2	1.5	1.0
3	2.7	3.0
3	2.5	3.0
3	1.0	1.0
3	1.0	0.0
4	2.3	2.0
4	3.3	3.5
4	2.1	2.0
4	1.7	1.0
5	2.5	2.0
5	1.2	1.0
5	1.0	1.0
5	2.3	2.5
6	2.5	2.0
6	2.0	2.0
6	4.5	4.0

Supplementary Table 1. Cartilage samples analyzed by paired histological and macroscopic score. Each sample was obtained from a different area of the indicated six donors.

S2

DONOR N. (1-9)	MACROSCOPIC SCORE (0-4)	EXPERIMENTAL CONDITION	
7	1	NC	Assay reproducibility
8	0	NC	
8	0	NC	Effect of compression and IL-1β stimulation
		C	
		NC+IL1-β	
		C+IL1-β	
9	0	NC	
		C	
		NC+IL-1β	
		C+IL-1β	

Supplementary Table 2. Cartilage samples analyzed by array-based gene expression analysis.

NC=not compressed
C=compressed (45min, 1Hz, 3MPa)

S3

Unigene	Refseq	Symbol	Description	2^(-Avg.(Delta(Ct))				Fold Change (comparing to NC NS)		
				NC	C	NC+IL1β	C+IL1β	C	NC+IL1β	C+IL1β
Hs.500483	NM_001613	ACTA2	Actin, alpha 2, smooth muscle, aorta	0.003258	neg	0.094732	neg		29.07	
Hs.118127	NM_005159	ACTC1	Actin, alpha, cardiac muscle 1	0.002562	neg	0.015463	neg		6.03	
Hs.369675	NM_001146	ANGPT1	Angiopoietin 1	0.003657	nv	nv	nv			
Hs.303649	NM_002982	CCL2	Chemokine (C-C motif) ligand 2	0.045279	0.025164	0.022956	neg	0.56	0.51	
Hs.251526	NM_006273	CCL7	Chemokine (C-C motif) ligand 7	0.004683	0.046633	neg	0.025693	9.96		5.49
Hs.592244	NM_000074	CD40LG	CD40 ligand	0.298334	0.052284	neg	0.064592	0.18		0.22
Hs.461086	NM_004360	CDH1	Cadherin 1, type 1, E-cadherin (epithelial)	0.003947	nv	nv	nv			
Hs.409662	NM_021110	COL14A1	Collagen, type XIV, alpha 1	0.016083	nv	nv	nv			
Hs.172928	NM_000088	COL1A1	Collagen, type I, alpha 1	0.080679	0.086419	0.014478	neg	1.07	0.18	
Hs.489142	NM_000089	COL1A2	Collagen, type I, alpha 2	0.150552	0.089158	neg	0.082042	0.59		0.54
Hs.443625	NM_000090	COL3A1	Collagen, type III, alpha 1	0.632148	0.289674	0.170164	0.281752	0.46	0.27	0.45
Hs.17441	NM_001845	COL4A1	Collagen, type IV, alpha 1	0.018304	nv	nv	nv			
Hs.570065	NM_000091	COL4A3	Collagen, type IV, alpha 3 (Goodpasture antigen)	nv	nv	nv	nv			
Hs.210283	NM_000093	COL5A1	Collagen, type V, alpha 1	0.017437	nv	nv	nv			
Hs.445827	NM_000393	COL5A2	Collagen, type V, alpha 2	0.057845	0.074197	0.025208	neg	1.28	0.44	
Hs.235368	NM_015719	COL5A3	Collagen, type V, alpha 3	0.003304	0.020369	neg	neg	6.17		
Hs.1349	NM_000758	CSF2	Colony stimulating factor 2 (granulocyte-macrophage)	0.002125	nv	nv	nv			
Hs.2233	NM_000759	CSF3	Colony stimulating factor 3 (granulocyte)	0.006052	0.224923	0.019038	0.071918	37.16	3.15	11.88
Hs.410037	NM_001901	CTGF	Connective tissue growth factor	0.424842	0.382227	0.503478	0.121371	0.90	1.19	0.29
Hs.712929	NM_001904	CTNNB1	Catenin (cadherin-associated protein), beta 1, 88kDa	0.009629	0.074713	0.009719	neg	7.76	1.01	
Hs.421724	NM_001911	CTSG	Cathepsin G	0.001660	nv	nv	nv			
Hs.632466	NM_000396	CTSK	Cathepsin K	0.034714	neg	0.087474	0.052284		2.52	1.51
Hs.610096	NM_001333	CTSV	Cathepsin L2	0.006066	nv	nv	nv			

Hs.789	NM_001511	CXCL1	Chemokine (C-X-C motif) ligand 1 (melanoma growth stimulating activity, alpha)	0.025886	neg	neg	0.041021			1.58
Hs.632592	NM_005409	CXCL11	Chemokine (C-X-C motif) ligand 11	0.001660	nv	nv	nv			
Hs.75765	NM_002089	CXCL2	Chemokine (C-X-C motif) ligand 2	0.027362	neg	0.022876	neg		0.84	
Hs.89714	NM_002994	CXCL5	Chemokine (C-X-C motif) ligand 5	0.015499	nv	nv	nv			
Hs.419815	NM_001963	EGF	Epidermal growth factor	0.005739	nv	nv	nv			
Hs.488293	NM_005228	EGFR	Epidermal growth factor receptor	0.011061	0.068039	0.019370	neg	6.15	1.75	
Hs.335513	NM_000129	F13A1	Coagulation factor XIII, A1 polypeptide	0.015250	0.066639	0.012648	neg	4.37	0.83	
Hs.62192	NM_001993	F3	Coagulation factor III (thromboplastin, tissue factor)	0.003206	nv	nv	nv			
Hs.351593	NM_000508	FGA	Fibrinogen alpha chain	0.002342	nv	nv	nv			
Hs.664499	NM_004465	FGF10	Fibroblast growth factor 10	0.003420	neg	neg	0.028213			8.25
Hs.284244	NM_002006	FGF2	Fibroblast growth factor 2 (basic)	0.106456	0.359111	0.158220	0.118052	3.37	1.49	1.11
Hs.567268	NM_002009	FGF7	Fibroblast growth factor 7	0.003533	0.019539	0.010784	neg	5.53	3.05	
Hs.799	NM_001945	HBEGF	Heparin-binding EGF-like growth factor	0.067218	0.233663	0.022483	0.044271	3.48	0.33	0.66
Hs.396530	NM_000601	HGF	Hepatocyte growth factor (hepapoietin A; scatter factor)	0.002804	nv	nv	nv			
Hs.856	NM_000619	IFNG	Interferon, gamma	0.005342	nv	nv	nv			
Hs.160562	NM_000618	IGF1	Insulin-like growth factor 1 (somatomedin C)	0.012502	neg	neg	0.056622			4.53
Hs.193717	NM_000572	IL10	Interleukin 10	0.004472	nv	nv	nv			
Hs.126256	NM_000576	IL1B	Interleukin 1, beta	0.010249	nv	nv	nv			
Hs.89679	NM_000586	IL2	Interleukin 2	0.003281	nv	nv	nv			
Hs.73917	NM_000589	IL4	Interleukin 4	0.003590	nv	nv	nv			
Hs.654458	NM_000600	IL6	Interleukin 6 (interferon, beta 2)	0.073218	0.058012	neg	0.040597	0.79		0.55
Hs.532082	NM_002184	IL6ST	Interleukin 6 signal transducer (gp130, oncostatin M receptor)	0.111749	0.150987	0.094078	neg	1.35	0.84	
Hs.644352	NM_181501	ITGA1	Integrin, alpha 1	0.023384	nv	nv	nv			
Hs.482077	NM_002203	ITGA2	Integrin, alpha 2 (CD49B, alpha 2 subunit of VLA-2 receptor)	0.045912	0.093915	neg	0.037747	2.05		0.82
Hs.265829	NM_002204	ITGA3	Integrin, alpha 3 (antigen CD49C, alpha 3 subunit of VLA-3 receptor)	0.019483	0.051922	neg	0.027537	2.67		1.41

Hs.440955	NM_000885	ITGA4	Integrin, alpha 4 (antigen CD49D, alpha 4 subunit of VLA-4 receptor)	0.001660	nv	nv	nv			
Hs.505654	NM_002205	ITGA5	Integrin, alpha 5 (fibronectin receptor, alpha polypeptide)	0.132893	0.131898	0.096055	0.041163	0.99	0.72	0.31
Hs.133397	NM_000210	ITGA6	Integrin, alpha 6	0.024097	0.201311	0.022718	neg	8.35	0.94	
Hs.436873	NM_002210	ITGAV	Integrin, alpha V (vitronectin receptor, alpha polypeptide, antigen CD51)	0.001660	0.047944	0.027299	0.029208	28.89	16.45	17.60
Hs.643813	NM_002211	ITGB1	Integrin, beta 1 (fibronectin receptor, beta polypeptide, antigen CD29 includes MDF2, MSK12)	0.156222	0.424107	0.031250	0.070928	2.71	0.20	0.45
Hs.218040	NM_000212	ITGB3	Integrin, beta 3 (platelet glycoprotein IIIa, antigen CD61)	0.041859	0.045673	0.013230	0.042320	1.09	0.32	1.01
Hs.13155	NM_002213	ITGB5	Integrin, beta 5	0.098641	0.132356	0.042541	neg	1.34	0.43	
Hs.470399	NM_000888	ITGB6	Integrin, beta 6	0.001660	0.029718	neg	neg	17.91		
Hs.431850	NM_002745	MAPK1	Mitogen-activated protein kinase 1	0.021271	0.068039	neg	neg	3.20		
Hs.861	NM_002746	MAPK3	Mitogen-activated protein kinase 3	0.013063	0.072670	0.031142	0.185244	5.56	2.38	14.18
Hs.407995	NM_002415	MIF	Macrophage migration inhibitory factor (glycosylation-inhibiting factor)	0.131063	0.165225	0.127185	0.047613	1.26	0.97	0.36
Hs.83169	NM_002421	MMP1	Matrix metallopeptidase 1 (interstitial collagenase)	0.084690	0.114427	0.079660	neg	1.35	0.94	
Hs.513617	NM_004530	MMP2	Matrix metallopeptidase 2 (gelatinase A, 72kDa gelatinase, 72kDa type IV collagenase)	0.087071	0.059643	0.028360	neg	0.68	0.33	
Hs.2256	NM_002423	MMP7	Matrix metallopeptidase 7 (matrilysin, uterine)	0.001660	nv	nv	nv			
Hs.297413	NM_004994	MMP9	Matrix metallopeptidase 9 (gelatinase B, 92kDa gelatinase, 92kDa type IV collagenase)	0.010537	nv	nv	nv			
Hs.535898	NM_002607	PDGFA	Platelet-derived growth factor alpha polypeptide	0.089312	0.111684	0.015093	neg	1.25	0.17	
Hs.491582	NM_000930	PLAT	Plasminogen activator, tissue	0.008229	0.029616	neg	neg	3.60		
Hs.77274	NM_002658	PLAU	Plasminogen activator, urokinase	0.001660	nv	nv	nv			
Hs.466871	NM_002659	PLAUR	Plasminogen activator, urokinase receptor	0.203298	0.143836	0.024775	0.033089	0.71	0.12	0.16
Hs.143436	NM_000301	PLG	Plasminogen	0.001660	nv	nv	nv			
Hs.729457	NM_000314	PTEN	Phosphatase and tensin homolog	0.025120	0.039899	0.015041	0.045201	1.59	0.60	1.80

Hs.196384	NM_000963	PTGS2	Prostaglandin-endoperoxide synthase 2 (prostaglandin G/H synthase and cyclooxygenase)	0.046552	0.104205	0.033726	0.060896	2.24	0.72	1.31
Hs.413812	NM_006908	RAC1	Ras-related C3 botulinum toxin substrate 1 (rho family, small GTP binding protein Rac1)	0.101649	0.228062	0.019777	neg	2.24	0.19	
Hs.247077	NM_001664	RHOA	Ras homolog gene family, member A	0.207570	0.119286	0.442884	0.226487	0.57	2.13	1.09
Hs.414795	NM_000602	SERPINE1	Serpin peptidase inhibitor, clade E (nexin, plasminogen activator inhibitor type 1), member 1	0.078111	0.148909	0.041378	0.071669	1.91	0.53	0.92
Hs.463059	NM_003150	STAT3	Signal transducer and activator of transcription 3 (acute-phase response factor)	0.028922	0.074197	0.073049	0.064369	2.57	2.53	2.23
Hs.410977	NM_003186	TAGLN	Transgelin	0.011801	0.069710	0.025916	neg	5.91	2.20	
Hs.170009	NM_003236	TGFA	Transforming growth factor, alpha	0.013033	nv	nv	nv			
Hs.645227	NM_000660	TGFB1	Transforming growth factor, beta 1	0.316805	0.232049	0.196827	0.090089	0.73	0.62	0.28
Hs.482390	NM_003243	TGFBR3	Transforming growth factor, beta receptor III	0.069589	0.084641	0.051119	0.036588	1.22	0.73	0.53
Hs.522632	NM_003254	TIMP1	TIMP metallopeptidase inhibitor 1	0.477973	0.974342	0.382889	0.292701	2.04	0.80	0.61
Hs.241570	NM_000594	TNF	Tumor necrosis factor	0.003281	0.020158	neg	neg	6.14		
Hs.73793	NM_003376	VEGFA	Vascular endothelial growth factor A	0.086870	0.314798	0.208050	0.074713	3.62	2.39	0.86
Hs.2257	NM_000638	VTN	Vitronectin	0.001660	nv	nv	nv			
Hs.492974	NM_003882	WISP1	WNT1 inducible signaling pathway protein 1	0.006208	neg	0.014131	neg	2.73	2.28	
Hs.643085	NM_003392	WNT5A	Wingless-type MMTV integration site family, member 5A	0.003549	nv	nv	nv			

Supplementary Table 3. Array-based gene expression analysis of OA cartilage samples. A focused panel of 84 genes involved in wound healing was analysed in two OA cartilage samples. Four experimental conditions were tested: 1) NC (not compressed cartilage, control group); 2) C (compressed cartilage); 3) NC+IL1-β (not compressed cartilage stimulated with the pro-inflammatory factor IL1-β); and 4) C+IL1-β (compressed cartilage stimulated with the pro-inflammatory factor IL1-β). 2^(-Avg. ΔCt) values are reported for the NC group, chosen as control group, while for other groups fold changes compared to NC are reported.

S4

Gene Symbol	C_t value										
	NC					C		NC+IL1β		C+IL1β	
	Don 7	Don 7*	Don 8	Don 8*	Don 9	Don 8	Don 9	Don 8	Don 9	Don 8	Don 9
ACTA2	30,23	29,91	≥35	32,08	≥35	≥35	≥35	27,66	≥35	≥35	≥35
ACTC1	31,4	32,34	neg	33,12	≥35	≥35	≥35	32,89	≥35	≥35	≥35
ANGPT1	33,51	33,36	31,58	37,66	≥35	≥35	≥35	36,65	39,63	≥35	≥35
CCL2	29,95	29,94	28,37	27,32	≥35	33,86	≥35	≥35	31,75	≥35	≥35
CCL7	36,39	39,58	32,72	33,03	34,76	32,08	≥35	≥35	≥35	37,57	34,31
CD40LG	≥35	≥35	12,53	≥35	≥35	≥35	31,75	≥35	≥35	≥35	31,65
CDH1	≥35	≥35	31,25	≥35	≥35	≥35	≥35	≥35	≥35	≥35	≥35
COL14A1	27,21	26,68	29,93	30,24	≥35	≥35	≥35	≥35	≥35	≥35	≥35
COL1A1	22,36	22	27,23	25,96	≥35	30,3	≥35	33,08	≥35	≥35	≥35
COL1A2	20,16	19,62	25,59	24,9	≥35	30,21	≥35	≥35	≥35	30,96	≥35
COL3A1	17,09	16,64	24,14	23,18	31,96	29,83	31,98	32,19	28,78	29,47	32,93
COL4A1	≥35	36,78	30,11	29,5	≥35	≥35	≥35	≥35	≥35	≥35	≥35
COL4A3	32,57	≥35	≥35	≥35	≥35	≥35	≥35	≥35	≥35	≥35	≥35
COL5A1	≥35	25,38	30,16	29,66	≥35	≥35	≥35	≥35	≥35	≥35	≥35
COL5A2	22,99	21,18	27,55	27,08	≥35	30,74	≥35	≥35	31,48	≥35	≥35
COL5A3	31,31	32,43	≥35	32,02	≥35	34,47	≥35	≥35	≥35	≥35	≥35
CSF2	33,38	38,1	≥35	33,93	≥35	≥35	≥35	≥35	≥35	≥35	≥35
CSF3	33,11	33,21	34,16	32,38	32,86	27,54	≥35	33,53	33,76	33,78	32,56
CTGF	33,13	22,55	24,4	22,82	33,78	26,01	35,83	27,98	29,86	29,83	≥35
CTNNB1	29,04	27,33	≥35	27,39	≥35	30,72	≥35	34,23	≥35	≥35	≥35
CTSG	32,2	37,54	≥35	≥35	≥35	≥35	≥35	≥35	≥35	≥35	≥35
CTSK	23,91	22,4	28,71	28,13	≥35	≥35	≥35	31,64	31,25	32,26	≥35
CTSV	30,74	≥35	32,29	32,1	≥35	≥35	≥35	≥35	≥35	≥35	≥35
CXCL1	≥35	≥35	29,24	28,87	≥35	≥35	≥35	≥35	≥35	32,96	≥35
CXCL11	≥35	38,71	≥35	≥35	≥35	≥35	≥35	≥35	≥35	≥35	≥35
CXCL2	≥35	≥35	29,08	28,79	≥35	≥35	≥35	33,55	33,21	≥35	37,81
CXCL5	34	37,08	30,14	30,19	≥35	≥35	≥35	≥35	≥35	≥35	≥35
EGF	34	32,06	31,57	33,06	≥35	≥35	≥35	≥35	≥35	≥35	≥35
EGFR	35,06	30,75	31,63	30,16	≥35	30,99	≥35	≥35	32,24	≥35	≥35
F13A1	27,66	28,86	30,84	29,56	≥35	31,05	≥35	≥35	33,47	≥35	≥35
F3	28,86	29,11	≥35	32,15	≥35	≥35	≥35	≥35	≥35	≥35	≥35
FGA	≥35	≥35	≥35	33,51	≥35	≥35	≥35	≥35	≥35	≥35	≥35
FGF10	≥35	32,74	≥35	31,87	≥35	≥35	≥35	≥35	≥35	34,04	≥35
FGF2	26,38	25,33	26,02	25,97	≥35	28,11	33,08	30,79	30,39	29,91	≥35
FGF7	36,27	32,13	35,57	31,73	≥35	34,59	≥35	≥35	33,93	≥35	≥35
HBEGF	32,59	29,96	27,89	28,2	32,89	29,55	32,88	≥35	31,81	≥35	32,74
HGF	≥35	34,59	≥35	32,73	≥35	≥35	≥35	≥35	≥35	≥35	≥35
IFNG	≥35	≥35	35,62	30,96	33,98	≥35	≥35	≥35	≥35	≥35	≥35
IGF1	33,87	28,86	30,5	30,76	≥35	≥35	≥35	≥35	≥35	32,03	≥35

IL10	36,82	31,76	33,42	32,29	≥35	≥35	≥35	≥35	≥35	≥35	≥35
IL1B	≥35	≥35	30,46	31,66	≥35	≥35	≥35	≥35	≥35	≥35	≥35
IL2	≥35	≥35	≥35	32,05	≥35	≥35	≥35	≥35	≥35	≥35	≥35
IL4	36,22	36,03	≥35	31,66	≥35	≥35	≥35	≥35	≥35	≥35	≥35
IL6	≥35	≥35	26,65	26,96	≥35	32,39	34,06	≥35	39,51	32,99	≥35
IL6ST	28,06	26,08	26,94	26,37	33,47	28,69	≥35	31,6	31,08	≥35	≥35
ITGA1	32,68	27,78	30,5	31,2	31,85	≥35	≥35	≥35	≥35	≥35	≥35
ITGA2	33,71	30,91	27,72	27,91	≥35	30,06	≥35	39,48	≥35	33,2	≥35
ITGA3	≥35	32,07	30,38	30,74	33,22	31,77	≥35	≥35	≥35	34,11	≥35
ITGA4	≥35	32,86	≥35	≥35	≥35	≥35	≥35	≥35	≥35	≥35	≥35
ITGA5	32,24	27,75	26,24	25,52	34,27	29,08	≥35	31,91	30,71	34,02	33,93
ITGA6	32,83	28,02	29,03	29,39	38,6	30,03	32,83	31,78	38,32	≥35	≥35
ITGAV	34,08	26,3	36,85	≥35	≥35	32	≥35	34,23	32,02	35,53	33,94
ITGB1	33,76	23,79	25,13	25,2	≥35	27,77	32,94	30,86	≥35	31,38	≥35
ITGB3	32,92	32,17	29,32	29,57	32,14	32,14	≥35	≥35	33,34	≥35	32,87
ITGB5	27,66	27,21	26,2	26,12	≥35	29,07	≥35	32,88	32,09	≥35	≥35
ITGB6	≥35	≥35	≥35	≥35	≥35	≥35	33,38	≥35	≥35	≥35	≥35
MAPK1	28,56	27,7	30,87	28,09	≥35	30,99	≥35	≥35	≥35	≥35	≥35
MAPK3	28,4	27,97	31,52	31,83	32,72	30,8	≥35	33,79	32,08	31,61	32
MIF	23,85	23,05	25,8	26,66	33,63	30,55	32,88	32,38	29,43	33,35	34,18
MMP1	≥35	31,94	26,57	26,41	≥35	29,49	≥35	32,84	30,32	≥35	≥35
MMP2	25,41	25,16	27,45	26,83	33,58	31,37	≥35	32,7	33,44	≥35	≥35
MMP7	33,75	33,53	≥35	≥35	≥35	≥35	≥35	≥35	≥35	37,37	≥35
MMP9	≥35	32,53	31,16	30,84	≥35	≥35	≥35	≥35	≥35	≥35	≥35
PDGFA	27,77	27,91	26,65	26,1	≥35	29,56	≥35	32,96	≥35	≥35	≥35
PLAT	≥35	≥35	31,6	31,47	≥35	33,39	≥35	≥35	≥35	≥35	≥35
PLAU	30,63	30,47	≥35	≥35	≥35	≥35	≥35	≥35	≥35	≥35	≥35
PLAUR	28,79	29,24	26,36	25,97	31,86	29,55	34,28	32,69	33,84	33,58	≥35
PLG	≥35	≥35	≥35	≥35	≥35	35,5	≥35	≥35	≥35	≥35	≥35
PTEN	26,32	25,81	29,57	28,67	≥35	32,53	≥35	≥35	32,97	32,68	≥35
PTGS2	30,59	30,15	28,25	27,32	≥35	29,76	≥35	32,53	33,11	31,82	≥35
RAC1	25,32	25,2	26,61	25,58	≥35	29,67	32,83	≥35	32,18	≥35	≥35
RHOA	23,07	22,29	25,82	25,05	33,23	29,37	≥35	30,11	28,1	30,21	32,82
SERPINE1	30,68	28,75	27,22	26,11	≥35	28,73	≥35	32,62	32,43	31,35	≥35
STAT3	26,52	25,57	30,35	27,28	≥35	30,74	≥35	32,53	30,88	31,66	≥35
TAGLN	29,03	31,1	30,74	30,77	≥35	30,92	≥35	33,26	33,14	≥35	≥35
TGFA	31,63	32,84	30,61	30,47	≥35	≥35	≥35	≥35	≥35	≥35	≥35
TGFB1	27,62	26,66	25,71	25,28	31,28	27,45	≥35	30,5	30,05	33,24	32,45
TGFBR3	24,69	24,06	27,71	27,66	33,46	30,36	≥35	33,43	31,01	33,29	
TIMP1	24,44	23,92	24,04	23,69	32,76	26,85	31,46	31,06	27,57	31,01	31,28
TNF	≥35	≥35	34,18	32,87	≥35	≥35	34,5	≥35	≥35	≥35	≥35
VEGFA	25,53	24,37	26,69	26,18	≥35	29,38	32,19	30,29	30,1	31,23	≥35
VTN	≥35	≥35	≥35	≥35	≥35	≥35	≥35	≥35	≥35	≥35	≥35

WISP1	28,33	28,14	33,11	31,18	≥35	≥35	≥35	≥35	33,15	≥35	≥35
WNT5A	32,57	31,74	≥35	31,71	≥35	≥35	≥35	≥35	≥35	≥35	≥35
ACTB	23,98	23,67	23,41	22,22	32,53	24,87	≥35	29,59	29,05	29,99	27,53
B2M	21,59	21,43	26,17	25,75	≥35	29,15	≥35	31,09	31,43	31,34	≥35
GAPDH	23,86	23,46	25,54	24,47	≥35	27,22	30,48	31,01	31,18	33,03	27,64
HPRT1	28,8	27,54	30,24	29,61	≥35	≥35	≥35	39,45	≥35	≥35	≥35
RPLP0	19,5	18,79	22,39	22,16	31,88	25,7	30,9	27,12	25,96	27,3	32,67

Supplementary Table 4. Array-based gene expression analysis of OA cartilage samples. C_t values relative to 84 genes involved in wound healing and 5 housekeeping genes are shown. Four experimental conditions were tested: 1) NC (not compressed cartilage, control group); 2) C (compressed cartilage); 3) NC+IL1-β (not compressed cartilage stimulated with the pro-inflammatory factor IL1-β); and 4) C+IL1-β (compressed cartilage stimulated with the pro-inflammatory factor IL1-β). * duplicates run to check for assay reproducibility.
